# Physical and psychological repercussions on Nursing workers’ health in COVID-19 units: A mixed-methods research study*

**DOI:** 10.1590/1518-8345.6669.4002

**Published:** 2023-11-03

**Authors:** Alexa Pupiara Flores Coelho Centenaro, Rosângela Marion da Silva, Gianfábio Pimentel Franco, Leticia Silveira Cardoso, Lílian Moura de Lima Spagnolo, Clarice Alves Bonow, Marta Cocco da Costa, Cassio Adriano Zatti, Kaliandra Gallina

**Affiliations:** 1 Universidade Federal de Santa Maria, Campus Palmeira das Missões, Palmeira das Missões, RS, Brasil.; 2 Universidade Federal de Santa Maria, Campus Sede, Santa Maria, RS, Brasil.; 3 Universidade Federal do Pampa, Campus Uruguaiana, Uruguaiana, RS, Brasil.; 4 Universidade Federal de Pelotas, Campus Pelotas, Pelotas, RS, Brasil.

**Keywords:** Nursing, Occupational Health, COVID-19, Mental Health, Pandemics, Hospital Units, Enfermería, Salud Laboral, COVID-19, Salud Mental, Pandemias, Unidades Hospitalarias, Enfermagem, Saúde do Trabalhador, COVID-19, Saúde Mental, Pandemias, Unidades Hospitalares

## Abstract

**Objective:**

to analyze how Nursing workers in COVID-19 hospital units perceive the physical and psychological repercussions of work on their health, as well as to identify the factors associated with their perceptions.

**Method:**

a parallel-convergent mixedmethods study conducted with 359 Nursing workers from COVID-19 units in seven hospitals. For the collection of quantitative data, a questionnaire containing sociodemographic and labor variables and related to perceptions of physical and psychological repercussions were used, and for qualitative data, semi-structured interviews were used. For the analysis, inferential statistics and thematic content analysis were used.

**Results:**

daytime workers, who had more than one employment contract and worked more than 41 hours/week perceived more moderate/intense physical repercussions, reporting overload and time off deficits. Nurses and CLT workers perceived psychological repercussions more moderately/intensely, mentioning managerial overload and job dissatisfaction. Women were 97% more likely to perceive physical repercussions and three times more likely to perceive psychological repercussions when compared to men, reporting household and family overloads.

**Conclusion:**

work and family overloads, intensified by the pandemic context, were associated with the intensity with which Nursing workers perceived physical and psychological repercussions.

## Abstract (es)

**Objetivo:**

analizar cómo los trabajadores de enfermería de unidades hospitalarias de COVID-19 perciben las repercusiones físicas y psicológicas del trabajo sobre su salud, e identificar los factores asociados a la percepción.

**Método:**

estudio de métodos mixtos de convergencia paralela realizado con 359 trabajadores de enfermería de unidades de COVID-19 en siete hospitales. Para la recolección de datos cuantitativos se utilizó un cuestionario que contiene variables sociodemográficas, laborales y relacionadas con la percepción de repercusiones físicas y psicológicas, y para los datos cualitativos se utilizaron entrevistas semiestructuradas. Para el análisis se utilizó estadística inferencial y análisis de contenido temático

**Resultados:**

os trabajadores diurnos, que tenían más de un trabajo y trabajaban más de 41 horas/semana, percibieron las repercusiones físicas como más moderadas/intensas, debido a la sobrecarga y falta de días libres. Los enfermeros CLT percibieron repercusiones psicológicas más moderadas/intensas, y las atribuyeron a la sobrecarga gerencial y la insatisfacción laboral. Las mujeres tenían 97% más probabilidades de percibir repercusiones físicas y tres veces más probabilidades de percibir repercusiones psicológicas, en comparación con los hombres, en referencia a la sobrecarga doméstica y familiar.

**Conclusión:**

las sobrecargas laborales y familiares, intensificadas por la pandemia, se asociaron con la intensidad con la que los trabajadores de enfermería percibieron las repercusiones físicas y psíquicas.

## Abstract (pt)

**Objetivo:**

analisar como os trabalhadores de enfermagem de unidades hospitalares COVID-19 percebem as repercussões físicas e psicológicas do trabalho em sua saúde e os fatores associados à sua percepção.

**Método:**

estudo de métodos mistos paralelo-convergente realizado com 359 trabalhadores lotados em unidades COVID-19 de sete hospitais. Para a coleta de dados quantitativos foram utilizados um questionário contendo variáveis sociodemográficas, laborais e relacionadas às percepções de repercussões físicas e psicológicas e, para a qualitativa, entrevistas semiestruturadas. Para a análise utilizou-se estatística inferencial e análise temática de conteúdo.

**Resultados:**

trabalhadores diurnos, que tinham mais de um vínculo empregatício e trabalhavam mais de 41 horas/semana perceberam as repercussões físicas como mais moderadas/intensas, devido à sobrecarga e déficit de folgas. Enfermeiros e celetistas perceberam as repercussões psicológicas mais moderadas/intensas, atribuindo-as à sobrecarga gerencial e insatisfação trabalhista. Mulheres apresentaram 97% mais chance de perceberem repercussões físicas e três vezes mais chance de perceberem repercussões psicológicas, quando comparadas aos homens, referindo sobrecarga doméstica e familiar.

**Conclusão:**

sobrecargas laborais e familiares, intensificadas pelo contexto pandêmico, foram associadas à intensidade com a qual os trabalhadores da enfermagem perceberam repercussões físicas e psicológicas.

**Highlights**

**(1)** Approximately half of the workers noticed physical repercussions and, most of them, psychological repercussions.

**(2)** Women were 97% more likely to perceive physical repercussions.

**(3)** Women presented three times more chance of perceiving psychological repercussions.

**(4)** Overload and physical repercussions were perceived in the day shift and with higher hour loads.

**(5)** Nurses and CLT workers perceived psychological repercussions and professional devaluation.

## Introduction

The COVID-19 pandemic represented a health crisis whose social, economic and health-related repercussions had no recent antecedents in the world. The individuals that developed the severe form of the disease required high-complexity clinical care. Health systems struggled to manage these demands, mobilizing human, financial, material and infrastructure resources. Among the challenges potentiated by the pandemic, the physical and psychological repercussions on health professionals who worked to cope with the disease stood out ^(^ 1 ^-^ 2 ^)^, comprising what was called the front-line. 

Front-line Nursing workers faced various adverse situations. Many professionals worked outside their specialization and training areas; others faced resource shortages and inadequacies in infrastructure in the face of the demand from critically-ill patients with significant mortality rates. The courses of action for managing COVID-19 patients were constantly updated. With human resources availability below the ideal, Nursing workers faces adversities that put their coping potential to the test ^(^ 2 ^)^. 

Nursing teams had to work in a context marked by overcrowding, overload and exhaustion ^(^ 3 ^)^. As a result, there is diverse evidence related to physical repercussions, such as contamination by SARS-CoV-2 ^(^ 4 ^)^, pain in various body parts (especially headache), dyspnea, dizziness, lethargy, nausea and skin lesions. These repercussions were mainly related to the working conditions and to intensive use of Personal Protective Equipment (PPE) items ^(^ 5 ^-^ 6 ^)^. 

The COVID-19 pandemic also affected Nursing workers’ mental health. It is known that the stress experienced in the front line was related to psychological repercussions such as anxiety and depression symptoms, sleep disorders and Burnout ^(^ 5 ^-^ 7 ^)^. In extreme situations, work can be associated with Post-Traumatic Stress Disorder, suicidal ideation and consummated suicide in Nursing ^(^ 8 ^)^. 

It is agreed that success in overcoming the COVID-19 pandemic lies in managing the repercussions on Nursing workers’ health ^(^ 8 ^)^. Workers’ Health Care needs to be implemented based on initiatives that promote well-being at work; however, their planning has to be evidence-grounded ^(^ 2 ^)^. Therefore, this study provides subsidies to understand how the perception of physical and psychological repercussions behaves according to the characteristics of this population group – elements that may guide health promotion actions at work. 

A literature review study pointed out the need to promote research studies exploring the interfaces between COVID-19 and physical and psychological repercussions in Nursing workers. Much has been produced in China (where the pandemic emerged) through cross-sectional research studies; however, it is necessary to deepen knowledge production in other countries ^(^ 6 ^)^ and through other methodological approaches, such as qualitative ones ^(^ 9 ^)^. 

To the present day, no surveys were identified that analyzed this study object according to the perception of workers themselves and in the light of mixed methodologies (qualitative and quantitative). In this sense, the objective of the current study was to analyze how Nursing workers in COVID-19 hospital units perceive the physical and psychological repercussions of work on their health, as well as to identify the factors associated with their perception.

## Method

### Type of study

A multicenter and parallel-convergent mixed-methods study, characterized by simultaneous and independent collection and analysis of qualitative and quantitative data, with equal weighing in the quantitative and qualitative stages (QUANT+QUAL) ^(^ 10 ^)^. Choice of this methodological design was based on the complex nature of the phenomenon under study, which required complementarity of different evidence sources to achieve deeper results. A correlational, cross-sectional and quantitative stage triangulation/combination was performed, concomitantly with a descriptive qualitative stage. The recommendations proposed in the Mixed Methods Appraisal Tool (MMAT) for methodological quality and transparency were adopted ^(^ 11 ^)^. 

### Setting

The research was conducted in seven hospitals from different municipalities in Rio Grande do Sul, Brazil. The criterion to choose these institutions was that they should be a reference in the State Contingency Plan for coping with COVID-19 in its respective meso-regions. Therefore, hospitals from the Metropolitan, Mid-East, Southern Campaign, Mid-West, Southeast and Northwest meso-regions were included (the latter included two hospitals, located in different cities).

Four were large-sized hospitals and three were medium-sized institutions. Five were philanthropic and two were public. Units classified as COVID-19 were included: one Respiratory Screening unit, five Urgency and Emergency sectors, four Clinical Inpatient units, and four Intensive Care Units (ICUs).

### Collection period and research team

The field stage took place between September 2020 and July 2021 (with the qualitative and quantitative collection procedures performed in parallel). The collection procedures were in charge of PhD in Nursing professors with due experience in qualitative and quantitative research. Two MSc students in Nursing also took part, previously trained for these activities.

### Population and selection criteria for the quantitative stage

The population was comprised by the Nursing workers (nurses, nursing technicians and nursing assistants) allocated to these units. Those who were on holiday or on any other type of work leave were excluded. The institutions forwarded to the research team the lists with the population that met the eligibility criteria, which totaled 470 individuals. Of these, 49 belonged to the Metropolitan meso-region; 25, to the Mid-East; 114, to the Northwest; 104, to the Southern Campaign; 47, to the Mid-West; and 131, to the Southeast. The study’s quantitative sample consisted of 359 nursing workers (76.4% of the eligible population). There were no losses related to the return of the instruments.

### Quantitative data collection

The quantitative stage was extended to the entire eligible population, in order to obtain the largest possible sample. A questionnaire containing the following variables was applied: sociodemographic: sex (male and female), age range (up to 33 years old and 34 years old and over), skin color (white and non-white), marital status (with partner and without partner), number of children (no children, one child and more than one child) and employment: job position (nurse or nursing technician), allocation unit (more than one COVID-19 unit, COVID-19 and non-COVID-19 unit, Another COVID-19 unit, COVID-19 Hospitalization, COVID-19 Urgency and Emergency and COVID-19 ICU), work shift (day, night and mixed), type of contract (Consolidation of Labor Laws - CLT, statutory, temporary, other), weekly workload (up to 40 hours, from 41 to 80 hours, greater than 80 hours), other employment relationship (yes/no) and time in the profession (less than five years, five years and more).

There were also two questions formulated as follows: “Do you perceive physical repercussions related to your work in COVID-19 hospital units?” and “Do you perceive psychological repercussions related to your work in COVID-19 hospital units?”. Both had the following alternatives: front-line work caused many (physical/psychological) repercussions on my health; front-line work caused moderate (physical/psychological) repercussions on my health; front-line work caused few (physical/psychological) repercussions on my health; and front-line work had no (physical/psychological) repercussions on my health. The participants had to indicate the sentence they deemed more synergistic with their perception.

This stage was conducted online in six hospitals. The questionnaire was prepared using Google Forms (a G Suite ^®^ tool), where the variables were preceded by the Free and Informed Consent Form (FICF) in full with the option to indicate agreement. The link to access the questionnaire was sent to the entire eligible population by email and/or via a messaging app, with mediation by the Nursing managers and hospital administrators. 

One of the institutions requested that the collection procedure be performed in-person. In this case, each worker was approached in their workplace by one of the research team members and was handed in an envelope containing the printed questionnaire, along with two copies of the FICF. Collection of the material was scheduled with each participant, according to their availability.

### Definition of the qualitative stage participants

Representativeness of all seven institutions in the qualitative stage was prioritized by selecting a group of five respondents *per* institution. All of them were selected in simple random draws, conducted with the aid of an online app ( https://app-sorteos.com/pt/apps/sorteio-de-nomes). Twelve workers failed to answer in this process, even if contacted three times. There were also five formal refusals attributed to work and research overload. In these cases (no answer or refusal), new draws were made until reaching five participants *per* institution. When all 35 interviews were finished, the material was submitted to a pre-analysis that identified theoretical data saturation ^(^ 12 ^)^, thus determining conclusion of this stage. 

### Qualitative data collection

The qualitative stage included conducting individual semi-structured interviews. In five hospitals, the interviews were conducted in-person, at the workplace and in comfortable, reserved and safe environments, observing all the biosafety measures. Two institutions requested that the interviews be conducted online. In these cases, the Google Meet digital platform was used (a tool from G Suite ^®^). The conversations were mediated by triggering questions that encouraged the participants to discuss their perceptions related to the physical and psychological repercussions of front-line work to cope with COVID-19. 

A first interview, considered as a pilot, was conducted; as there was no need to implement any adjustment in the question script, it was included in the database. The interviews were audio-recorded with the participants’ consent and lasted approximately 22.5 minutes.

It is noted that all 35 participants in the qualitative stage also answered the online questionnaire; therefore, they were included in the quantitative stage. This verification was made when conducting the semi-structured interviews.

### Quantitative and qualitative data treatment and analysis and data triangulation/combination

The quantitative analysis was performed by tabulating and coding the data in an Excel spreadsheet and later on transferring them to the SPSS statistical software, version 20.0. The missing data, as well as the variables with data below the minimum sample population or above 10%, were not considered in the analysis.

Mean values and absolute (n) and relative (%) frequencies were used to describe the sample. The questions referring to the perceptions about the physical and psychological repercussions were selected as dependent variables. In order to ease the analysis, the answers were dichotomized intro “reduced or null perception” and “moderate or intense perception”. Moderate or intense perceptions of physical and psychological repercussions were associated with sociodemographic and work-related characteristics (independent variables), by means of Fisher’s Exact and Pearson’s Chi-square tests. The statistical significance level adopted was 5%. In addition to that, associations between independent variables and the outcomes of interest were estimated by calculating the Odds Ratios, with their corresponding 95% Confidence Intervals.

The qualitative analysis adopted the thematic content analysis procedures: pre-analysis, exploration of the material, and treatment and interpretation of the data obtained ^(^ 13 ^)^. In the pre-analysis, the pertinent material to meet the study objective was selected, which was submitted to floating and comprehensive reading. Exploration of the material involved breaking down the text into Registration Units (RUs) with the aid of the NVivo software. The RUs were organized by semantic affinity and grouped in two analytical axes presented in the results of this study. 

Treatment and interpretation of the data obtained took place concomitantly with data triangulation/combination. The findings from the different evidence sources were integrated by merging qualitative and quantitative data, which, after being analyzed independently, were compared to find complementarities or differences, in a convergent design. To accomplish this integration, “joint-display” strategies were used, that is, visual representations that illustrate QUAL+QUANT findings and are characterized by the visual presentation of triangulation/combination in mixed-methods designs ^(^ 14 ^)^. The CmapTools software, version 6.04 was used to such end; it is a tool that allows creating mental and conceptual maps. 

### Ethical aspects

When presenting the qualitative findings, the respondents are identified by the letter W (for “Worker”), followed by the characterization of gender (female or male) and age in years old. The ethical precepts set forth in resolutions No. 466/2012 and No. 510/2016 of the National Health Council were observed. The project was approved in 2020 by a local Research Ethics Committee under Protocol No. 4,206,065. Additionally, Amendments No. 4,363,162 and 4,395,923 (both in 2020) were approved by the same Ethics Committee, allowing flexibility of in-person and online collection procedures, according to the institutions’ internal policies and to the course of the pandemic.

## Results

A total of 359 Nursing workers took part in this research (76.4% of the eligible population). There was predominance of individuals self-identified as women (n=278, 85%), with a mean age of 33 (±12.42) years old, white-skinned (n=260, 79.5%), and married or in stable unions (n=175, 53.5%). Regarding job position, nursing technicians prevailed (n=250, 76.4%), bound to the Consolidation of Labor Laws ( *Consolidação das Leis do Trabalho*, CLT) regime (n=212, 64.8%) and with weekly hour loads of up to 40h (n=200, 61.1%). In the sample under study, 37.6% (n=135) were allocated to COVID-19 ICUs; 18.7% (n=67), to COVID-19 Inpatient Units; 11.7% (n=42), to COVID-19 Respiratory Screening units or to Urgency and Emergency units; 11.7% (n=42) reported being allocated to other COVID-19 units (with different names, but with similar work processes); and 7% (n=25) were allocated to more than one COVID-19 sector. 

Among the 359 workers who participated in the study, 45.1% (n=162) had moderate or intense perception of work-related physical repercussions in COVID-19 hospital units, whereas 54.9% (n=197) had reduced or null perceptions. In relation to the psychological repercussions, 53.8% (n=193) perceived them as moderate or intense, whereas 46.2% (n=166) had reduced or null perceptions.

In the semi-structured interviews, the physical and psychological repercussions proved to be mutually related and linked to the stress and overload inherent to the COVID-19 units, as pointed out in the testimonies: *[...] I would eat because of anxiety. I noticed some weight gain. Awful sleep quality. Three o’clock, four o’clock in the morning I wake up on alert. Constant headache. It’s stress. A frustrating sensation for trying to manage the patient and not being able to [...] we lost many patients here [...] I gradually saw the need to see her [psychologist]. Because I wasn’t able to cope with it. I don’t sleep any more. Anxiety, eating, headache, stomach ache, gastritis. I didn’t have that before. It’s a stress thing. [...] We’re reaching exhaustion, and from there to depression is but a leap [...] (W9, F, 39 years old). [...] every now and then a colleague gets sick, depressed or has COVID-19 symptoms. Many had already been distanced. At the beginning of the pandemic I was on work leave, but I believe that it’s because of these issues [psychological], because in the middle of the afternoon I had shortness of breath, headache, chest pain, but it was my mind that did it. We’re doing ourselves a lot of harm. We leave here exhausted, with symptoms that were not supposed to happen. It’s normal to be tired, but the psychological part has to be OK [...] (W26, F, 39 years old)*

**Figure 1 -:**
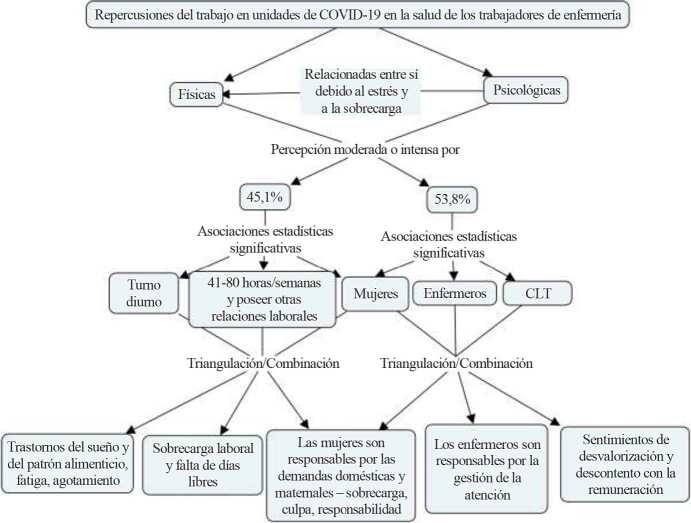
Joint display illustrating the QUAL+QUANTI triangulation/combination of findings related to the physical and psychological repercussions and associated factors among Nursing workers in COVID-19 hospital units. Rio Grande do Sul, Brazil, 2020-2021

Figure 1 illustrates the joint-display representing the qualitative and quantitative data integration. Both analysis axes that discuss the study findings will be presented below. 

### Perceptions about the physical repercussions and their interface with sociodemographic and work-related aspects among Nursing workers from COVID-19 hospital units

Associations were sought between the perceptions about physical repercussions and the sociodemographic and work-related variables. The analysis results suggest that: mixed-shift workers were 60% less likely to perceive moderate or intense physical repercussions when compared to day workers (p=0.003); workers with hour loads between 41 and 80 hours *per* week were 73% more likely to perceive moderate or intense physical repercussions when compared to those who worked up to 40 hours *per* week (p=0.025); those who had more than one employment contract were 52% more likely to perceive moderate or intense physical repercussions when compared to those who had only one job (p=0.031); and those who identified themselves as women were 97% more likely to perceive moderate or intense physical repercussions when compared to those who identified themselves as men (p=0.017). These findings are presented in Table 1: 

**Table 1 -:** Perceptions about psychological repercussions associated with sociodemographic and work-related variables among Nursing workers from COVID-19 hospital units (n=359). Rio Grande do Sul, Brazil, 2020-2021

Sociodemographic and work-related variables		Perceptions about physical repercussions				OR (IC95%)*	p-value †
		Reduced or null		Moderate or intense			
		n ‡	%	n ‡	%		
Gender	Female	158	80,2	144	88,9	1,97 (1,08; 3,60)	0,017 §
	Male	39	19,8	18	11,1	1,0	
Age group	Up to 33 years old	72	41,86	53	37,32	1,0	0,414 §
	34+ years old	100	58,14	89	62,68	1,20 (0,76; 1,90)	
Skin color	White	157	79,7	126	77,8	1,0	0,376 §
	Not white	40	20,3	36	22,2	1,12 (0,67; 1,86)	
Marital status	With a partner	84	42,6	84	51,9	1,0	0,051 §
	Without a partner	113	57,4	78	48,1	0,69 (0,45; 1,04)	
Children	No children	67	34,0	56	34,6	1,0	0,543 §
	One child	57	28,9	39	24,1	0,81 (0,47; 1,40)	
	More than one child	73	37,1	67	41,4	1,09 (0,67; 1,78)	
Job position	Nurses	42	21,3	45	27,8	1,0	0,155 §
	Nursing Technicians	155	78,7	117	72,2	0,70 (0,43; 1,14)	
Allocation unit	More than one COVID-19 unit	66	33,5	69	42,6	1,29 (0,62; 2,66)	0,170 §
	COVID-19 and NON-COVID-19 units	28	14,2	14	8,6	0,61 (0,24; 1,52)	
	Other COVID-19 units	33	16,8	34	21,0	1,27 (0,57; 2,82)	
	COVID-19 hospitalization unit	21	10,7	17	10,5	1,0	
	COVID-19 Urgency and Emergency units	15	7,6	10	6,2	0,82 (0,29; 2,29)	
	COVID-19 ICU||	34	17,3	18	11,1	0,65 (0,27; 1,54)	
Work shift	Day	67	34,0	66	40,7	1,0	0,00 ^3^§
	Night	73	37,1	73	45,1	1,01 (0,63; 1,62)	
	Mixed	57	28,9	23	14,2	0,40 (0,22; 0,74)	
Type of contract	CLT ¶	133	67,0	108	66,7	0,99 (0,60; 1,64)	0,406 **
	Statutory	45	22,8	37	22,8	1,0	
	Other	2	1,0	6	3,7	3,64 (0,69; 19,15)	
	Temporary	17	8,6	11	6,8	0,83 (0,34; 2,02)	
Hour load	Up to 40	142	72,1	95	58,6	1,0	0,025 §
	41-80 hours	43	21,8	50	30,9	1,73 (1,07; 2,81)	
	More than 80 hours	12	6,1	17	10,5	2,11 (0,96; 4,63)	
Another employment contract	No	119	60,4	81	50,0	1,0	0,03 ^1^§
	Yes	78	39,6	81	50,0	1,52 (1,0; 2,32)	
Time in the profession	Less than 5 years	72	36,5	53	32,7	1,0	0,259 §
	5+ years	125	63,5	109	67,3	1,18 (0,76; 1,83)	

* OR (95%CI) = Odds Ratio and 95% Confidence Interval.

† p = Probability;

‡ n = Absolute frequency;

§ Pearson’s Chi-square test;

|| ICU = Intensive Care unit;

¶ CLT =*Consolidação das Leis do Trabalho* (Consolidation of Labor Laws);

** Fisher’s Exact test

The qualitative findings contributed to understanding the overload that existed among day shift workers, with two employment contracts and overlapping working hours, due to the overload and the time off deficits felt by these groups of workers. The physical repercussions manifested themselves as changes in sleep, eating pattern, fatigue and exhaustion. In addition to that, the women’s feeling of accountability in relation to the household and maternal demands was verified, which represented yet another overload factor. The triangulation/combination of these findings with the statistically significant associations can be seen in Figure 2: 

**Figure 2 -:** QUAL+QUANT triangulation/combination related to the perceptions about physical repercussions on the health of Nursing workers from COVID-19 hospital units. Rio Grande do Sul, Brazil, 2020-2021

Moderate or intense perceptions about physical repercussions on health		
Day shift (p*=0.003)	Hour loads from 41 to 80 hours (p*=0.025) and having another employment contract (p*=0.031)	Female gender (p*=0.017)
[...] [day] *it’s six hours. It’s complicated because there’s only one day off during the week. One Sunday per month, it’s the law. All the other days I’m here: weekends, Fridays, Saturdays, Sundays. It becomes heavy. “12 x 36” would be ideal. I even imagined that it might change because in the “in and out” inside the COVID-19 unit, we’re taking it* [contamination] *out.. Leaving it “12 by 36” would be better for us, as it is at night* […] *I don’t sleep enough time, sometimes I don’t eat what I want. I just can’t do any physical activity* [...] (W†2, M. ‡, 39 years old)	*[...] after I graduated, I always had two jobs. I’m already used to that routine. But I know that the body reacts* [...] (W†2, M‡. 39 years old) […] *I worked in two places and I go to college* […] *but the routine was really heavy, so I resigned and stayed only in the hospital* […] *but I still do “extras” as an instrumentator, because of the financial issue* [...] *I work a lot and study, I live tired. I sometimes dream about the ICU§* [...] *I wake up very early in the morning, and I sometimes wake up already tired. It seems that my brain just can’t disconnect* [...] (W †29, M. ‡, 42 years old)	[…] *I try to clear my head, do some physical activity, keep my mind busy. But I have a son that needs my attention. I give attention, but I see that I feel exhausted* [...] (W †6, F. ||, 24 years old) [...] *I’m a lioness for my children* [...] *the biggest priority in my life is them* [...] *I think: “I can’t be weak, I have to be fine for them”. Sometimes I get home willing to do nothing. I want to take a bath, lie down in the dark, wake up the next day. But it’s not my reality. I need to recover because I have things to do at home* [...] (W †9, F. ||, 39 years old)

* p = Probability;

† W = Worker;

‡ M = Male;

§ ICU = Intensive Care Unit;

|| F = Female

### Perceptions about the psychological repercussions and their interface with sociodemographic and work-related aspects among Nursing workers from COVID-19 hospital units

When associating the perceptions about psychological repercussions with sociodemographic and work-related variables it was identified that: nursing technicians were 54% less likely to perceive moderate or intense psychological repercussions when compared to nurses (p=0.003); workers hired via CLT, also called CLT workers, were twice as likely to perceive moderate or intense psychological repercussions when compared to their statutory counterparts (p=0.004); and those who identified themselves as women were three times more likely to perceive moderate or intense psychological repercussions when compared to those who identified themselves as men (p<0.001). Table 2 shows these findings: 

**Table 2 -:** Perceptions about psychological repercussions associated with sociodemographic and work-related variables among Nursing workers from COVID-19 hospital units (n=359). Rio Grande do Sul, Brazil, 2020-2021

Sociodemographic and work-related variables		Perceptions about psychological repercussions				OR (IC95%)*	Valor p†
		Reduced or null		Reduced or null			
		n ‡	%	n ‡	%		
Gender	Female	126	75,9	176	91,2	3,28 (1,78; 6,05)	<0,001 §
	Male	40	24,1	17	8,8	1,0
Age group	Up to 33 years old	51	36,43	74	42,53	1,0	0,272 §
	34+ years old	89	63,57	100	57,47	0,77 (0,49; 1,22)	
Skin color	White	130	78,3	153	79,3	1,0	0,824 §
	Not white	36	21,7	40	20,7	0,94 (0,56; 1,56)	
Marital status	With a partner	79	47,6	89	46,1	1,0	0,780 §
	Without a partner	87	52,4	104	53,9	1,06 (0,70; 1,60)	
Children	No children	54	32,5	69	35,8	1,0	0,406 §
	One child	50	30,1	46	23,8	0,72 (0,42; 1,23)	
	More than one child	62	37,3	78	40,4	0,92 (0,60; 1,60)	
Job position	Nurses	28	16,9	59	30,6	1,0	0,003 §
	Nursing Technicians	138	83,1	134	69,4	0,46 (0,27; 0,76)	
Allocation unit	More than one COVID-19 unit	59	35,5	76	39,4	1,04 (0,50; 2,15)	0,239 §
	COVID-19 and NON-COVID-19 units	25	15,1	17	8,8	0,55 (0,22; 1,33)	
	Other COVID-19 units	25	15,1	42	21,8	1,36 (0,60; 3,05)	
	COVID-19 hospitalization unit	17	10,2	21	10,9	1,0	
	COVID-19 Urgency and Emergency units	14	8,4	11	5,7	0,63 (0,23; 1,75)	
	COVID-19 ICU||	26	15,7	26	13,5	0,80 (0,34; 1,87)	
Work shift	Day	62	37,3	71	36,8	1,0	0,073 §
	Night	59	35,5	87	45,1	1,28 (0,80; 2,06)	
	Mixed	45	27,1	35	18,1	0,67 (0,38; 1,18)	
Type of contract	CLT ¶	99	59,6	141	73,1	2,11 (1,26; 3,52)	0,004 **
	Statutory	49	29,5	33	17,1	1,0	
	Other	1	0,6	7	3,6	10,39 (1,22; 88,45)	
	Temporary	17	10,2	12	6,2	1,18 (0,49; 2,85)	
Hour load	Up to 40	115	69,3	122	63,2	1,0	0,429 §
	41-80 hours	40	24,1	53	27,5	1,24 (0,97; 2,02)	
	More than 80 hours	11	6,6	18	9,3	1,54 (1,06; 3,40)	
Another employment contract	No	87	52,4	113	58,5	1,0	0,243 §
	Yes	79	47,6	80	41,5	0,77 (0,51; 1,18)	
Time in the profession	Less than 5 years	50	30,1	75	38,9	1,0	0,083 §
	5+ years	116	69,9	118	61,1	0,67 (0,43; 1,05)	

* OR (95%CI) = Odds Ratio and 95% Confidence Interval.

† p = Probability;

‡ n = Absolute frequency;

§ Pearson’s Chi-square test;

|| ICU = Intensive Care Unit;

¶ CLT =*Consolidação das Leis do Trabalho* (Consolidation of Labor Laws);

** Fisher’s Exact test

The qualitative findings contributed to understanding the mental exhaustion that existed among nurses, as they are a reference in their teams and due to the institutional demands, as well as among CLT workers, due to labor dissatisfactions (such as wage devaluation and little recognition). Women also proved to be oftentimes distressed by the demands of motherhood in the work-related stress context, and also by the fear of being contamination vectors for their families. The triangulation/combination of these findings with the statistically significant associations can be seen in Figure 3: 

**Figure 3 -:** QUAL+QUANT triangulation/combination related to the perceptions about psychological repercussions on the health of Nursing workers from COVID-19 hospital units. Rio Grande do Sul, Brazil, 2020-2021

**Moderate or intense perceptions about psychological repercussions on health**		
Nurses (p*=0.003)	Women (p*<0.001)	Consolidation of Labor Laws (p*=0.004)
[...] *it’s very busy because I’m the only nurse for everything. My team has nine technicians, so there are nine technicians calling me all the time, plus doctors, plus patients, plus the routines I have to do as a nurse. It’s busy and stressful* [...] (W †11, F ‡. 30 years old) [...] *We’re under a lot of pressure for things that are not our fault. It’s a cultural thing. Because we’re on the front line, we’ve always been. Nurses are always in the spotlight and under pressure. We’re the people responsible for care* [...] (W †12, F ‡. 40 years old)	[...] *we have to be able to prioritize and give attention* [to the family] *but I sometimes end up taking it* [work-related stress] *home. Sometimes I’m on the run, my children come to me, but I keep saying: “I’ll get back to you, mom’s coming” and I can’t go* [...] (W †9, F ‡. 39 years old) [...] *I’m a mother. I have a baby at home. I also have my father with cancer living with me. I’m really afraid, I can’t get contaminated. I work tormented* [...] (W †32, F ‡. 41 years old)	[...] *Nursing is not valued as it should. Nursing technicians need three jobs to provide a decent living for their family* [...] *We risk our lives for low wages* (W †5, M §. 36 years old) [...] *we’re not valued. Our wage is not very good. We work Saturdays and Sundays, holidays, Christmas Eve, New Year’s Eve* [...] (W †12, F ‡. 40 years old) [...] *very outdated*[wage]. *You have to work in two, three places to get paid a little better* (W †29, M §. 42 years old)

* p = Probability;

† W = Worker;

‡ F = Female;

§ M = Male

## Discussion

Working with COVID-19 patients has potentiated physical and psychological ailments in Nursing workers due to the high contamination risk, but also due to the physical and psychological burdens of the front line routine ^(^ 15 ^)^. The moderate or intense perceptions of psychological harms were greater than those of physical harms, which corroborates the results of an international literature review study showing that. among various productions related to the health of front-line Nursing workers coping with the pandemic, the psychological repercussions were more reported than the physical ones, as a consequence of the stressful routine in COVID-19 units ^(^ 6 ^)^. 

On the other hand, the qualitative findings suggest that the physical and psychological repercussions are mutually related as a response to stress and overload in the front line. COVID-19 had repercussions on nurses’ health and workforce, as a consequence of occupational risks, worsened by precarious working conditions, insufficient supplies and the health crisis. Nursing workers had to develop strategies to care for themselves and their teams while managing the repercussions of the pandemic on their personal lives and families ^(^ 16 ^)^. Therefore, working in the front line imposed a new reality on the workers, whose challenges oftentimes exerted impacts on their physical and psychological health. 

The physical repercussions were directed at a group of workers comprised by those who had more than one job, worked more than 41 hours *per* week, were allocated to the day shift (characterized by daily shifts, including holidays and weekends), and women (historically related to double or triple working hours). This points to a profile: people who experience overload from more than one job, whose shifts took up a large part of their days, and which also added to their family demands. 

A Brazilian study showed that, during the COVID-19 pandemic, Nursing workers faced exhausting work characterized by an increase in number of activities and a reduction in rest time. This situation included the pressure exerted by the pandemic on family and domestic life, especially for women; it was also reinforced by precarious working conditions and by the professional weakening of Nursing, influenced by low wages and labor flexibilization – a reflection of a Neoliberal conjuncture. Under the Neoliberal policy auspices, the COVID-19 pandemic imposed an upsurge in precarious work, influencing Nursing workers’ health ^(^ 17 ^)^. 

It is important to discuss each factor that is associated with the physical repercussions. The data suggest that mixed-shift workers were 60% less likely to experience moderate or intense physical repercussions when compared to day workers. This result is different from other studies conducted with Nursing workers, which have evidenced physical and psychological repercussions related to night work ^(^ 18 ^-^ 19 ^)^. However, the qualitative data help explain this by showing that, in the exclusive daytime shift, the schedules were organized in almost daily shifts, which were extended on weekends and holidays, increasing the frequency of workers’ exposure to COVID-19 and the feeling of work overload. 

A literature review study investigated the repercussions of shift-work on the health and life of workers in general. The authors argue that extended shifts are the only negative variable. Working hours that extend over weekends, holidays in general and festive dates have negative repercussions on workers’ social, family and marital relationships, weakening important dimensions of their lives ^(^ 20 ^)^. 

It is also important to highlight that COVID-19 characterized an important increase in the workload for Nursing, rendering work shifts more complex. This was added to a social distancing context, more intensely experienced by the front-line workers due to the fear to transmit the virus to their family members ^(^ 21 ^)^. Therefore, it can be considered that, at some moments, working in COVID-19 units absorbed the professionals, generating certain imbalance between their work and personal life. In these cases, the moderate/intense perceptions about physical repercussions can result from the combination of work overload, stress and dissatisfaction. 

Workers working between 41 and 80 hours *per* week were 73% more likely to experience moderate or intense physical repercussions compared to those working up to 40 hours *per* week. In addition to that, those with more than one employment contract were 52% more likely to experience moderate or intense physical repercussions when compared to those with only one job. The qualitative findings suggest that individuals who worked in more than one place (and consequently doubled their hour load) felt overworked, tired and also perceived physical repercussions. 

Double working hours is a recurring issue in Nursing. One of the causes is the influence of the Neoliberal model on the hiring regimes, which is why the profession is oftentimes not financially profitable, causing workers to seek more than one employment contract to obtain material retributions from their work. It is also known that Nursing work organization in shifts allows people to reconcile activities in two or more places, which is why this has become an almost cultural practice among workers ^(^ 22 ^)^. 

A Chinese study conducted with Nursing workers active during the COVID-19 pandemic evidenced an association between longer workdays and higher fatigue scores ^(^ 18 ^)^. Increased working hours and/or overlapping jobs were intensified with COVID-19 due to high team contamination, absenteeism and workforce retention difficulties. Longer workdays are described as an element that potentiates overload and increases the chances of physical repercussions ^(^ 7 ^)^. 

Regarding the psychological repercussions, they were more intensely perceived by the group comprised by nurses, CLT workers and women. It is important to individually analyze the factors that proved to be associated with the psychological repercussions.

When compared to nurses, nursing technicians had 54% fewer chances of perceiving moderate or intense psychological repercussions. The qualitative findings evidenced nurses’ overload in terms of care management. A mixed-methods study showed an association between suspicion of Common Mental Disorders and the “Nurse” professional category; these professionals identified important work pressures related to care management ^(^ 23 ^)^, which is close to these findings. 

Care management implies mobilizing various activities and decisions that allow nurses to lead their teams. It requires knowledge and physical and emotional skills that prepare each professional to act in various adverse situations ^(^ 24 ^)^. The COVID-19 pandemic increased the complexity inherent to Nursing care management. In addition to increased demand and work overload, nurses were involved in reorganizing units and teams, forecasting and providing more supplies, establishing new routines, protocols and training ^(^ 25 ^)^. 

Nurses who were in charge of managing the units, in particular, had to face the impacts of the pandemic and ensure availability of supplies, reinforce communication strategies and sustain new work process protocols ^(^ 16 ^)^. Therefore, in addition to the clinical pressure of caring for critically-ill COVID-19 patients, nurses had to manage a changing work process, which potentiated their mental overload. 

Workers who identified as women were 97% more likely to perceive physical repercussions and three times more likely to perceive moderate or intense psychological repercussions when compared to those who identified as men. In the interviews, it was noticed that, at various moments, the female group highlighted family and maternal demands as an element that potentiated their overload, which can be related to gender roles that oftentimes encourage women to take responsibility for household care.

The results of a research study conducted with hospital Nursing workers evidenced that women presented more physical repercussions when compared to men ^(^ 19 ^)^. There is diverse evidence that the COVID-19 pandemic has especially affected the health of young women with children, which can be a consequence of the social impacts imposed by the pandemic ^(^ 7 ^-^ 23 ^)^. 

Research studies with health and nursing workers who were active in COVID-19 evidenced associations between the gender variable (women) and higher anxiety scores ^(^ 24 ^)^ and showed that women had worse mental health outcomes when compared to men ^(^ 26 ^)^. 

In their testimonies, people who identified themselves as women also showed self-responsibility, thinking that they were not sufficiently meeting the affective demands of their children, and also worrying about the risk of contaminating them with SARS-CoV-2. Other research studies confirm that health and nursing workers who were active in the pandemic had important concerns about the well-being and health of their families, especially fearing to be COVID-19 transmission vectors ^(^ 1 ^,^ 3 ^,^ 7 ^,^ 27 ^)^. 

A literature review study investigated aspects related to Nursing workers’ mental health during the COVID-19 pandemic. One of the outstanding elements was the influence of gender issues on the profession. The Nursing workforce has historically been mostly female. Socially, the work of caring for others goes beyond the working hours and is projected into the family space, in relationships with children and other loved ones ^(^ 28 ^)^. 

Finally, it was found that the CLT participants were twice as likely to perceive moderate or intense psychological repercussions when compared to statutory employees. The qualitative findings suggest that this can be a reflection of the pay gap, causing feelings of devaluation.

In Brazil, the CLT legislation, responsible for regulating the laws governing employment contracts, has undergone changes in its laws, aiming to meet Neoliberal political projects; the result was loss or flexibilization of various labor rights. It is known that there are weaknesses in CLT and that, because of this, Nursing workers are sometimes vulnerable to occupational risks, overloaded and without proper financial compensations. It is projected that, in the coming years, Nursing workers’ dissatisfaction may influence emptying of the profession, similarly to what is already the case in some world countries ^(^ 29 ^)^. 

In a study with Nursing workers in the COVID-19 context, they were asked about the main elements that demotivated them to work. In the sample under study, 64% mentioned remuneration; 54% working hours; and 40% insufficient labor benefits ^(^ 30 ^)^. Another survey, also conducted with Nursing workers during COVID-19, showed that remuneration, benefits and conditions for promotions emerged as some of the main factors of job dissatisfaction ^(^ 31 ^)^. 

A survey conducted with female Nursing workers during COVID-19 showed that they felt that the presence of the State in guaranteeing the labor rights of the category was diminishing. They reinforced the demand for a wage floor and 30 hours *per* week during this period ^(^ 17 ^)^, highlighting that wage and working hours issues are factors that permeate Nursing workers’ mental health in most of the Brazilian hospital institutions. 

It is important to consider that the influence of Neoliberalism in Brazil on Nursing workers (especially on those who do not have the stability of a public job position, such as CLT workers) is a reality prior to the pandemic. There is a cycle of setbacks in social labor rights, where some guaranteed workers’ rights have recently been removed and retirement rules have been changed for a significant part of the population. Consequently, it is reinforced that the Neoliberal logic in the organization of health work produces wear out and illness in Nursing workers ^(^ 17 ^)^. 

In addition, it is noted that the Nursing workers who comprised the front line should be carefully monitored, based on institutional actions to recognize the repercussions of work on their health and to mitigate the consequences that can be inherited in the long-term ^(^ 9 ^)^. It becomes necessary to recognize the factors associated with the physical and psychological repercussions of front-line work to support occupational health promotion actions in the post-pandemic period, during which institutions will need a healthy workforce engaged in reorganizing the health system. 

In addition to that, it is of utmost importance that Nursing workers are duly recognized for their coping efforts throughout the health crisis. Promoting occupational health and professional appreciation and mitigating the physical and psychological repercussions of front-line work should be an investment by institutions and society as a way to recognize of the importance of Nursing in Brazil.

This study adds diverse information that strengthens the historical record of how the COVID-19 pandemic intersected with the path of Brazilian Nursing. This research highlights the elements that potentiated the repercussions of the COVID-19 pandemic on the physical and mental health of those who served on the front line. It is known that all workers were exposed to physical and psychological stressors. However, with this study it is possible to reinforce the analysis at the group of workers comprised by of women, nurses and CLT workers, who accumulated working hours and jobs and who added many shifts, as it is suggested that they represented especially vulnerable groups. During the post-pandemic period, these groups will require special care, supported by research, so that illness does not become a legacy of the front-line performance.

The limitation of this study is the time period during which the data were collected. Over a year, the collection procedures went through different phases of the pandemic, characterized by curves of greater contagion (and consequently greater pressure on health services), intersected by moments of greater pandemic control and the beginning of vaccination of health workers. It was important to extend collection throughout this period to reach an encompassing response rate and to systematize the interviews. In addition to that, transit in the COVID-19 sectors and collection in the online modality represented challenges that culminated in a longer period of time in the field stage. Therefore, it is important to consider that the interviewees’ answers may reflect the different moments that characterized this time frame.

## Conclusion

Physical repercussions were perceived moderately or intensely by 45.1% of the workers, especially by daytime ones, who had more than one employment contract and worked more than 41 hours/week, who reported overload and time off deficits. Psychological repercussions were perceived moderately or intensely by 53.8% of the workers, especially nurses and CLT workers, who reported managerial overload and work dissatisfaction. Women were 97% more likely to perceive physical repercussions and three times more likely to perceive moderate/intense psychological repercussions when compared to men, reporting an overload of household and maternal demands. Therefore, it can be concluded that work and family overloads, intensified by the pandemic context, were associated with the intensity with which Nursing workers perceived physical and psychological repercussions.
